# Summary of the Course of Demonstrative Surgery in the University of Pennsylvania, from October 9th to December 12th

**Published:** 1851-01

**Authors:** 


### CLINICAL REPORTS.

---

*Summary of the course of Demonstrative Surgery in the University of Pennsylvania, from October 9th to December 12th.*

The lectures given under the above title at the University of Pennsylvania are so designated, in order to distinguish them from the Clinical Instruction furnished at the Pennsylvania Hospital. This course differs from hospital instruction in the patients being usually presented to the student only in the lecture room, whilst the points of pathology, surgical anatomy, &c., connected with the case, are illustrated by preparations and drawings from the Wistar Museum. The title therefore seems sufficiently distinctive. Under the system of practical teaching now pursued by the Faculty, the student has opportunities of seeing not only the severe cases of the wards of an hospital, but also the tumors, operations and lighter cases of ordinary practice. He is therefore amply instructed in practical details; being first engaged for two hours on each Wednesday and Saturday at the Pennsylvania Hospital, and then occupied for two hours more, in witnessing the operations and demonstrations of practical surgery and medicine, within the University building.

The Demonstrative Surgical Lectures are conducted by Dr. Gibson, Professor of Surgery, on Wednesday at 1 o'clock, and by Dr. Wm. E. Horner, Professor of Anatomy, on Saturday, at the same hour; both being aided by Dr. Henry H. Smith, assistant Lecturer on Demonstrative Surgery. In addition to the bi-weekly lectures, the student can also follow the daily treatment of patients in the Dispensary of the Institution, where much of the office business of the physician and surgeon is amply illustrated. A male and female ward, capable of containing the cases of severe operation, together with a well assorted pharmacy, complete the arrangements of the faculty for conducting this part of their instruction. Experience (as tested by the increased attendance of both practitioners and students) has proved that these lectures are a most valuable addition to the former system of clinical instruction, and as the large number of students resorting to the Pennsylvania Hospital during the Winter months, renders it impossible for the class to visit the wards, every addition to their facilities for the observation of disease, must be regarded as possessing increased value.

The surgical demonstrations being those which are especially adapted to this kind of teaching, we have collected, as illustrative of the general character of the course, a condensed statement of the more important cases shown since the opening of the session of 1850—51.

*Oct. 9th*, 1850. Dr. Gibson commenced the lectures by some introductory remarks on the plan at present pursued by the medical faculty in reference to practical instruction; alluding to his having first originated it many years since, when, owing to the defects of the alms-house hospital, it became necessary for him to add to the clinical instruction of his class. Since then, the more perfect arrangements of that hospital had rendered his system unnecessary until within the last five years, when similar circumstances had again required its renewal. As it had been now well tested, he thought that its value might be considered as established, not as presenting a substitute for hospital instruction, for on that the faculty had always insisted and materially contributed to, in the hospital course, but as affording the class additional means for the practical study of surgery, and especially of such cases as they would first meet with in practice, yet seldom see in a hospital.

*Amputation of the Leg.*

After these remarks, and an explanation of the arrangements of the dispensary, &c., Dr. Smith brought forward a patient who had labored for fifteen months under necrosis, &c., of the tibia, from a compound fracture of the leg, caused by being run over by a rail road car, and who was now being exhausted by hectic. The Dr. therefore amputated the leg below the knee, the patient being so well etherised, as to be perfectly unconscious of pain throughout the operation, and having a smile on his face during the dressing. The Anæsthetic agent now, and for some time, employed in the University, consists of four parts of washed Ether to one of Chloroform, the latter being found to prevent the muscular excitement occasionally seen, where ether alone is employed, whilst the extreme sedation of the chloroform is obviated by the ether. The stump being subsequently dressed with the cold water dressing, did well.

*Amputation of Half of the Lower Jaw.*

On the 16th, Dr. Gibson presented to the class a patient suffering from a large osteo-sarcoma of the lower maxilla, for which he performed amputation of one half of the bone, disarticulating and removing it without ligating any vessel of importance. This patient was also etherized, but not perfectly, lest the escape of blood into his throat should cause strangulation. Six weeks subsequently the man was again shown, laboring under a salivary fistula, and with evident signs of a renewal of the disease.

*Dislocation of the Elbow, reduced after six weeks.*

On the 19th, Dr. Horner presented a girl who had been laboring for several weeks under a dislocation of both bones of the forearm, backwards, at the elbow. This patient being etherized whilst the pullies were in action, and their power thus applied without resistance, was soon relieved of the deformity; after which an angular splint was employed, the angle being changed occasionally so as to prevent stiffness of the joint. The Doctor also removed a toe-nail without the patient's being at all conscious of it. The extreme pain attending this operation, as formerly practiced, illustrated fully the benefits of etherisation. In removing the nail, the operator explained the advantages derived from freeing it from the matrix, and removing it entire, instead of the usual operation of eversion after an incision through its middle; which, although it removed the cause of the disease at the time, yet left the patient exposed to the growth of a new nail. This mode has also been lately advocated by a surgeon in Tours.

*Removal of the Parotid Gland.*

On the 26th, Dr. Horner operated for the removal of the parotid gland in a gentleman from the South, assisted by Drs. Smith, J. Neill, and J. H. B. McClellan. This patient had been laboring under a carcinoma of the gland for more than a year; and the tumor was now about the size of the hand loosely closed, but not yet softened. Though perfectly aware of the risk of a return of the disease, Mr. O'B. preferred the chances of recovery, to the endurance of the violent neuralgic pains caused by the pressure of the tumor upon the nerves of the face. After the application of the anæsthetic, the Doctor made a crucial incision over the tumor, the lines of incision being freely continued along the base of the jaw, and also down the neck in the course of the carotid artery. On turning back the flaps, the tumor was found to be firmly bound down by fibres of the platysma-myodes muscle, and fascia of the neck. After dividing these freely, the dissection was commenced from behind, the tumor having contracted firm adhesions to the anterior edge of the sterno-cleido-mastoid muscle. By working carefully forwards, the gland, which was surrounded by a firm capsule formed by the effects of the inflammation upon the cellular substance around it, was gradually freed from its posterior and inferior attachments, when the primitive carotid being brought fairly into view, was found to have been so much involved in the disease as to show very considerable thickening of its coats, giving it the form and distended appearance of the vessel when injected in the subject. To guard against sudden hemorrhage, a ligature was therefore passed around the artery on a level with the larynx, but not tied. The dissection being again resumed, the upper and anterior attachments of the tumor were divided, and the primitive carotid being tied, the tumor was removed from its deep attachments. These were, here, not so deep as in the healthy condition, the adhesion of the tumor to the angle of the jaw having favored its exit from the deepest points. The division of the internal maxillary having caused considerable recurrent hemorrhage, the internal and external carotids were also tied, lest in their patulous condition, recurrent hemorrhage should ensue through them also. The submaxillary gland being evidently involved in the disease, it was removed, as well as a chain of diseased lymphatics leading towards and adhering to the thyroid on the same side. The wound now made was a perfect gap, and exposed freely all the deep parts of this region; but, on a close examination of the neck, it was impossible to find either the internal jugular vein or the *par vagum* of this side, the probability being that the pressure caused by the binding down of the tumor by the fascia, &c., had caused their disappearance. The operation lasted fifty-five minutes, during most of which the patient inhaled the anæsthetic, although he was not fully etherised during the entire operation. The wound was then filled with lint, and the flaps loosely confined by sutures. On the *eighth* day all the ligatures had separated spontaneously, and a long thin slough showed all that remained of the upper portion of the carotid on this side. About six weeks afterwards the wound had filled up considerably, but was not allowed to close, a surface of three inches by one and a half or two inches, being purposely kept open in the hopes of doing good by the suppuration. The patient then started for home, in charge of his medical attendant, who was requested to dress the part with acetic acid, in order, if possible, to destroy any diseased growth that might appear.

*Resection of the Femur for Deformed Fracture.*

Nov. 2d. Dr. Horner brought forward a man, from Schuylkill county, who had received a fracture of his femur, eighteen months previous, but which had been allowed to unite with great deformity, the bone being bent angularly on the outer side of the thigh, and the limb shortened to the extent of four inches.

Having etherised the patient very thoroughly, Dr., Horner assisted by Drs. Smith, Neill, and Chas. Evans, made an incision on the outside and back of the thigh, so as to avoid the blood-vessels, and give vent to any subsequent suppuration, and then sawed off the two oblique ends so as to make the fracture transverse. The pullies being now applied, in order to overcome the shortening, powerful extension was made, and the fragments slipped past each other, so as to bring the limb straight, and elongate it to within half an inch of its proper length. The two fresh surfaces of bone being thus brought in contact, Physick's long splints were applied, together with circular splints around the seat of fracture, and the patient was put to bed. For 36 hours he did well, but mortification of the limb quickly showing itself, he became typhoid, and, notwithstanding the most active treatment, sank rapidly, and died four days after the operation. From its being impossible to obtain an autopsy, the cause of death could not be positively determined, but the impression was, that the mortification resulted from phlebitis, caused either by some injury to the veins, during the extension, or from their compression against the callus surrounding the fracture. A careful external examination showed that the femoral vein was thickened, or filled with a clot, as far up as the external iliac, whilst the limb, from the knee to the iliac region of the same side, was emphysematous and mottled. Pressure in the groin forced gas out of the wound, from which there also issued a considerable amount of dark grumous blood, which possibly had resulted from hæmorrhage beneath the fascia femoris, but which did not show itself externally, in consequence of the splint closing the wound.

*Exostosis of Nasal Bones.*

On Nov. 9th Dr. Horner removed an exostosis as large as a large chesnut from the nasal bones of a man, but the patient was unable to give any history of its origin.

*Abscess in the Cancellated Structure of the Tibia.*

He also presented a patient with an enlargement of the tibia, at its lower third, of about three months duration, the result of a blow. Believing it to be a case of osteitis, and that the excruciating pain was due to an abscess in the cancellated structure of the tibia, the Doctor laid bare the bone, and perforating it with a trephine, so as to expose the structure near the medullary cavity, obtained nearly a half ounce of pure and apparently healthy pus, thus verifying most satisfactorily the diagnosis. The patient was quickly relieved, and in about ten days returned to his home.

*Extirpation of the Left Testicle.*

On the 16th, a patient, laboring under a fungus of the left testicle, was presented by Dr. Horner, and the history of the case fully detailed. After the administration of the anæsthetic, the testicle was removed in the usual manner, the arteries of the cord being separated from the vas deferens, before the application of the ligature, or the division of the cord. The wound being then united by sutures, was subsequently dressed with cold water, and did well.

*Amputation of the Mammary Gland.*

A case of well marked scirrhus of the breast, unaccompanied by axillary disease or ulceration, was brought forward, on the 23d of November, by Dr. Smith. After detailing the history and peculiarities of the case, the Doctor etherised the patient and removed the entire gland, without the woman's exhibiting any consciousness of suffering. After completing the dressing, allusion was made, by Doctor Smith, to a point of practice suggested to him by Dr. Horner, viz.: the importance of avoiding, if possible, any removal of the fascia, or sheath of the pectoral muscle; it having been proved in several cases, that its injury impaired the free motion of the arm, especially in such movements as put the pectoralis major upon the stretch. Two weeks subsequently, this patient was again exhibited, improved in health, and with the wound rapidly cicatrizing.

### *Fungus of the Dura Mater.*

On Nov. 27th, Dr. Gibson presented a boy, æt. 13 years, who had upon the left half of the os frontis, near the left frontal protuberance, a small tumor about the size of a horse-chesnut, or buck-eye. This tumor was elastic, partially moveable, and in most respects similar in appearance to the ordinary atheromatous encysted tumor. It was said to have existed from childhood, and for several years was quite small, but within the last nine months it had rapidly increased. A close examination proved it, however, to be something of a more serious character than was at first supposed, as gentle pressure caused its partial disappearance, although it did not produce any cerebral symptoms, whilst an opening with a sharp well defined edge, could be distinctly felt in the os frontis, by an accurate and experienced finger. Having therefore little doubt as to its being a fungus of the dura mater, the Doctor cautioned the class in regard to a mistake in the diagnosis of such tumors, and the extreme danger that would result should an operation be attempted.

A similar tumor, but of much larger size, was presented at the Dispensary, during the summer, by Dr. Horner. This, on being tested with a needle, bled freely, and was immediately closed,—but the patient has since been lost sight of. The importance and rare character of these tumors therefore induce the publication of this record of them.

### *Dislocation of the Lens and Staphyloma.*

A child suffering from this injury, and also one of Hare Lip, were presented and operated on by Dr. Horner, on Nov. 30th, after which a suspicious tumor was removed from the arm of an old lady, who had been suffering for eighteen months, under a disease believed, by Dr. Smith, to be that of moluscum.

### *Artificial Limbs.*

After some remarks by Dr. Horner on Coxalgia, in connection with an acute case shown Dec. 7th, Dr. Smith exhibited the patient whose leg he had amputated October 9th, and explained the advantages to be derived from artificial limbs, both in the upper and lower extremities. Those shown seemed to be well adapted to the purpose, and the mechanism of a hand was especially good, it enabling the patient under favorable circumstances to grasp a hat, whip, &c. After referring to the importance of attention, on the part of the surgeon, to the prevention of ulceration of the stump, as well as to the length, when it is proposed to apply an artificial limb, he mentioned that those then shown were made by Messrs. Kretchmar & Gildea, No. 64 Dock street, and had several points to recommend them. He also exhibited a well marked case of Rickets, in which the limbs had been very much straightened by the use of Rohrer's splints.

A case of umbilical hernia and one of fistula lachrymalis were also exhibited by Dr. Horner.

In addition to the above summary of cases, many other patients, suffering under interesting surgical complaints, have been shown at various times, and the peculiarities of their complaints thoroughly explained by the lectures. The general character of these remarks, together with such other points of future cases as may possess general interest, will be hereafter reported somewhat more in detail.

